# Sub-threshold spinal cord stimulation facilitates spontaneous motor activity in spinal rats

**DOI:** 10.1186/1743-0003-10-108

**Published:** 2013-10-24

**Authors:** Parag Gad, Jaehoon Choe, Prithvi Shah, Guillermo Garcia-Alias, Mrinal Rath, Yury Gerasimenko, Hui Zhong, Roland R Roy, Victor Reggie Edgerton

**Affiliations:** 1 Biomedical Engineering IDP, University of California, Los Angeles, CA 90095, USA; 2 Neuroscience IDP, University of California, Los Angeles, CA 90095, USA; 3 Department of Integrative Biology and Physiology, University of California, Los Angeles, Terasaki Life Sciences Building, 610 Charles E. Young Drive East, Los Angeles, CA 90095-7239, USA; 4 Neurobiology, University of California, Los Angeles, CA 90095, USA; 5 Neurosurgery, University of California, Los Angeles, CA 90095, USA; 6 Brain Research Institute, University of California, Los Angeles, CA 90095, USA; 7 Pavlov Institute of Physiology, St. Petersburg 199034, Russia

**Keywords:** Sub-threshold spinal cord stimulation, Spinal cord injury, Spontaneous cage activity, EMG, Motor control

## Abstract

**Background:**

Epidural stimulation of the spinal cord can be used to enable stepping on a treadmill (electrical enabling motor control, eEmc) after a complete mid-thoracic spinal cord transection in adult rats. Herein we have studied the effects of eEmc using a sub-threshold intensity of stimulation combined with spontaneous load-bearing proprioception to facilitate hindlimb stepping and standing during daily cage activity in paralyzed rats.

**Methods:**

We hypothesized that eEmc combined with spontaneous cage activity would greatly increase the frequency and level of activation of the locomotor circuits in paralyzed rats. Spontaneous cage activity was recorded using a specially designed swivel connector to record EMG signals and an IR based camcorder to record video.

**Results and conclusion:**

The spinal rats initially were very lethargic in their cages showing little movement. Without eEmc, the rats remained rather inactive with the torso rarely being elevated from the cage floor. When the rats used their forelimbs to move, the hindlimbs were extended and dragged behind with little or no flexion. In contrast, with eEmc the rats were highly active and the hindlimbs showed robust alternating flexion and extension resulting in step-like movements during forelimb-facilitated locomotion and often would stand using the sides of the cages as support. The mean and summed integrated EMG levels in both a hindlimb flexor and extensor muscle were higher with than without eEmc. These data suggest that eEmc, in combination with the associated proprioceptive input, can modulate the spinal networks to significantly amplify the amount and robustness of spontaneous motor activity in paralyzed rats.

## Background

A wide range of animal spinal cord injury models and species have shown that stimulation applied to spinal neural networks can dramatically improve motor ability, i.e., enhance the ability to stand and step on a treadmill with partial body weight support [1-6]. More recently three completely paralyzed human subjects (one classified as ASIA A and two as ASIA B) were implanted with a commercially available spinal cord electrode array and stimulation package originally designed for pain suppression [7,8]. Epidural stimulation of specific spinal segments (via caudal electrodes at ~ S1 spinal level), in combination with the sensory information from the lower limbs and weeks of stand training, was sufficient to generate full weight-bearing standing. These subjects also recovered some voluntary control of movements of the toe, ankle, and the entire lower limb, but only when electrical enabling motor control (eEmc) was present. Thus, one possibility is that modulation of the excitability of the lumbosacral region of the spinal cord via eEmc, combined with the weak excitatory activity of descending axons that were not otherwise detectable, could volitionally achieve a level of excitation that was sufficient to activate the spinal motor circuits above the motor thresholds of a significant number of motoneurons among synergistic motor pools. These results in human subjects demonstrate that some patients clinically diagnosed as having complete paralysis can use proprioceptive information combined with some input from descending motor signals (perhaps residual but functionally silent without eEmc) to activate spinal motor circuits, thus generating and controlling a range of motor functions via eEmc.

There is some spontaneous activity in the paralyzed muscles after a complete mid-thoracic spinal cord transection. For example, the total amount of integrated EMG activity in the soleus and lateral gastrocnemius muscles in spinal cats during a 24-hr period was ~25% and ~33%, respectively, of that occurring in uninjured cats [9]. The present experiment was designed to determine the feasibility of enhancing the amount of spontaneous cage activity of paralyzed muscles using sub-threshold intensities of stimulation via chronically implanted epidural electrodes placed over the lumbosacral spinal cord in adult spinal rats. We chose rats that had experienced a rehabilitation process to step on a treadmill for 6 weeks under the influence of eEmc because chronic step training engages and reinforces the locomotor networks that would potentially be activated during spontaneous cage activity. We determined the activity levels and movement patterns of the hindlimbs of rats having a complete spinal cord transection at a low thoracic level while in their home cages during 6-hr periods with and without continuous eEmc (40 Hz). We hypothesized that eEmc would modulate the spinal locomotor circuits such that the hindlimbs would be more active during periods with than without eEmc. This would have the effect of more frequently engaging those neural networks that control the routine, spontaneous postural and locomotor functions that are critical in defining the level of functionality after severe paralysis. In general, the results are consistent with this hypothesis.

## Methods

### General animal procedures

Data were obtained from 4 adult female Sprague Dawley rats (270-300 g body weight). Pre- and post-surgical animal care procedures have been described in detail previously [10]. The rats were housed individually with food and water provided *ad libitum*. All survival surgical procedures were conducted under aseptic conditions and with the rats deeply anesthetized with isoflurane gas administered via facemask as needed. All procedures described below are in accordance with the National Institute of Health Guide for the Care and Use of Laboratory Animals and were approved by the Animal Research Committee at UCLA.

### Head connector and intramuscular EMG electrode implantation

A small incision was made at the midline of the skull. The muscles and fascia were retracted laterally, small grooves were made in the skull with a scalpel, and the skull was dried thoroughly. Two amphenol head connectors with Teflon-coated stainless steel wires (AS632, Cooner Wire, Chatsworth CA) were securely attached to the skull with screws and dental cement as described previously [3,10]. Selected hindlimb muscles, i.e., the tibialis anterior (TA) and soleus (Sol), were implanted bilaterally with EMG recording electrodes as described by Roy et al. [11]. Skin and fascial incisions were made to expose the belly of each muscle. Two wires extending from the skull-mounted connector were routed subcutaneously to each muscle. The wires were inserted into the muscle belly using a 23-gauge needle and a small notch (~0.5-1.0 mm) was removed from the insulation of each wire to expose the conductor and form the electrodes. The wires were secured in the belly of the muscle via a suture on the wire at its entrance into and exit from the muscle belly. The proper placement of the electrodes was verified during the surgery by stimulating through the head connector and post-mortem via dissection.

### Spinal cord transection and eEmc electrode implantation procedures and post-surgical animal care

A partial laminectomy was performed at the T8-T9 vertebral level. A complete spinal cord transection to include the dura was performed at approximately the T8 spinal level using microscissors. Two surgeons verified the completeness of the transection by lifting the cut ends of the spinal cord and passing a glass probe through the lesion site. Gel foam was inserted into the gap created by the transection as a coagulant and to separate the cut ends of the spinal cord.

For eEmc electrode implantation, partial laminectomies were performed to expose the spinal cord at spinal levels L2 and S1. Two Teflon-coated stainless steel wires from the head connector were passed under the spinous processes and above the dura mater of the remaining vertebrae between the partial laminectomy sites. After removing a small portion (~1 mm notch) of the Teflon coating and exposing the conductor on the surface facing the spinal cord, the electrodes were sutured to the dura mater at the midline of the spinal cord above and below the electrode sites using 8.0 Ethilon suture (Ethicon, New Brunswick, NJ). Two common ground (indifferent EMG and stimulation grounds) wires (~1 cm of the Teflon removed distally) were inserted subcutaneously in the mid-back region. All wires (for both EMG and eEmc) were coiled in the back region to provide stress relief.

All incision areas were irrigated liberally with warm, sterile saline. All surgical sites were closed in layers using 5.0 Vicryl (Ethicon, New Brunswick, NJ) for all muscle and connective tissue layers and for the skin incisions in the hindlimbs and 4.0 Ethilon for the back skin incision. All closed incision sites were cleansed thoroughly with saline solution. Analgesia was provided by buprenex (0.5–1.0 mg/kg, s.c. 3 times/day). The analgesics were initiated before completion of the surgery and continued for a minimum of 3 days. The rats were allowed to fully recover from anesthesia in an incubator. The rats were housed individually in cages that had ample CareFresh bedding, and the bladders of the spinal rats were expressed manually 3 times daily for the first 2 weeks after surgery and 2 times daily thereafter. The hindlimbs of the spinal rats were moved passively through a full range of motion once per day to maintain joint mobility. All of these procedures have been described in detail previously [5].

### Stimulation and testing procedures

The rats went through a bipedal step training rehabilitation process (20 min a day, 5 days a week) for 6 weeks under the influence of eEmc at 40 Hz between L2 and S1 at an intensity just above threshold [3] using a body weight support system [12]. Chronic step training was used because it engages and reinforces the locomotor networks that would potentially be activated during spontaneous cage activity.

The rats were tested under two conditions with and without eEmc at 40 Hz between L2 and S1 at 6 weeks post-injury: 1) during bipedal stepping on a specially designed motor-driven rodent treadmill using a body weight support system [4,5,13,14] and 2) during spontaneous cage activity. The eEmc during treadmill locomotion was set just above threshold as described previously [3]. The threshold for eliciting a muscle twitch and corresponding time linked EMG response (soleus was used as the reference muscle) was between 1.8 to 2 V for all rats. The sub-threshold level then was set at 20% below the motor threshold, i.e., between 1.4 and 1.6 V, during the recording of spontaneous cage activity.

The spontaneous activity levels of the spinal rats were determined in their home cage. The head connector was connected via cables to a set of amplifiers and a stimulator. A swivel arrangement was attached to the cables near the head connector to allow the rats to move freely in the cage. Food (pellets, pieces of fruit, and fruit loops) was distributed throughout the cage floor to encourage movement and exploration. Video data were recorded using a camcorder with a series of IR LEDs to enable recording in the dark, i.e., the active period for the rats. EMG data were amplified and recorded using custom LabView-based data acquisition software with a sampling frequency of 10 kHz. Data were recorded for 6 continuous hours starting at 8:00 pm and ending at 2:00 am. EMG recordings from the hindlimb muscles were band-pass filtered (1 Hz to 5 KHz), amplified using an A-M Systems Model 1700 differential AC amplifier (A-M Systems, Carlsborg, WA), and sampled at a frequency of 10 KHz using a custom data acquisition program written in the LabView development environment (National Instruments, Austin, TX) as described previously [5].

### Data analysis

The energy in the EMG signal for both muscles was calculated by estimating the area under the curve after rectification of the raw EMG (integrated EMG) as previously described [10,15,16]. The amounts of integrated EMG per one-min periods of stepping and spontaneous cage activity were compared. The EMG responses during spontaneous cage activity were binned in 1-min snippets for detailed analysis. A frequency distribution was constructed by estimating the energy within each 1-min bin and joint probability distributions to show the relationship between the activity of the soleus and TA were plotted. Video data were analyzed to estimate the total amount of time that the rats were active (mobile) in their home cages during the 6-hr recording period.

### Statistical analyses

All data are reported as mean ± SEM. Statistically significant differences were determined using paired t-tests. The criterion level for the determination of a statistical difference was set at *P* < 0.05 for all computations.

## Results

### Evidence of enabling vs. inducement of neuromuscular activity

**Figure 1:**
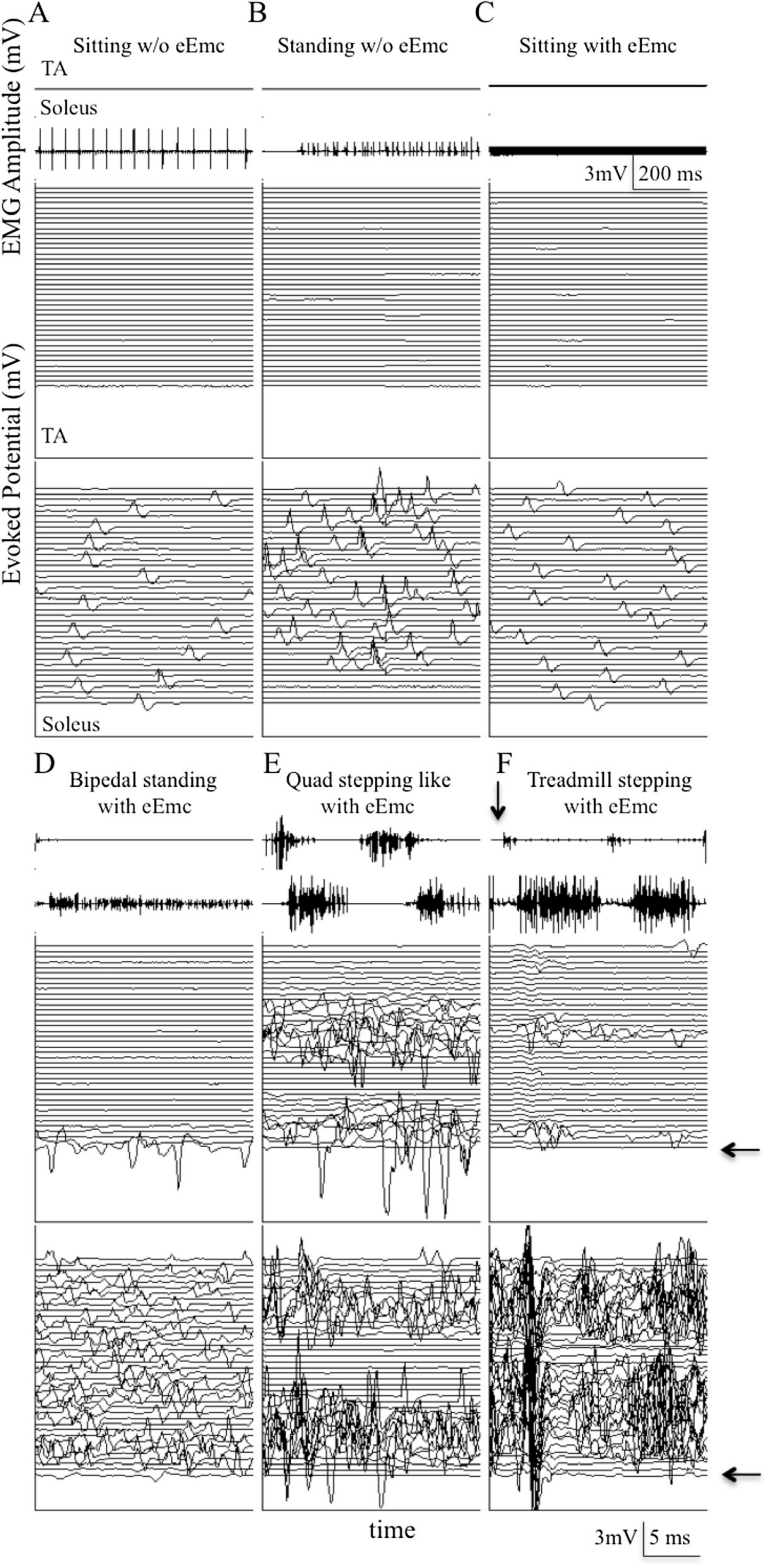
**Representative EMG and evoked potentials with and without eEmc.** Representative raw EMG and evoked potentials from the soleus and tibialis anterior (TA) muscles without eEmc from one spinal rat during **(A)** sitting, **(B)** attempted bipedal standing, and with eEmc (1.5 V, 40 Hz between L2 and S1) during **(C)** sitting, **(D)** bipedal standing, and **(E)** quadrupedal (Quad) stepping-like movement during the 6-hr recording period in its home cage. **(F)** Representative EMG and evoked potential from the soleus and TA from the same rat during body weight supported bipedal treadmill stepping facilitated by eEmc (2.0 V, 40 Hz between L2 and S1). The start of each trace with eEmc is synchronized with the initiation of the eEmc pulse. Each trace is 25 msec, i.e., the time between successive eEmc pulses. The arrow placed on the EMG signals denotes the time of the initial 25 msec scan.

We carefully examined the relationship between the absence or presence of eEmc and the amount and pattern of spontaneous cage activity. In the absence of eEmc there were periods of spontaneous activity when the rats remained in a sitting posture (Figure 1A) and on some occasions when it appeared that they were attempting to stand (Figure 1B) (Additional file 1: Video 1). EMG activity increased, particularly in the soleus, during incidences of apparent attempted standing (Figure 1B). The most common observed position was for the rats to have their hindlimbs completely extended often showing little or no movement except some spastic-like reactions. Even during movement propelled by the forelimbs, the upper body remained low with the head close to the floor of the cage and the hindlimbs extended.

A sub-motor threshold intensity of eEmc is evident by the absence of any time-linked evoked muscle responses (Figure 1C). In the presence of eEmc the forelimbs were used to move around in the cage more often than in its absence. During this activity the hindlimbs usually dragged behind showing some bursting in both the flexor and extensor muscles (Figure 1E) and the upper body was maintained at a greater height compared with that seen without eEmc. The rats often would stand on the hindlimbs with partial weight bearing using the sides of the cage as support (Figure 1D, Additional file 2: Video 2), a behavior never observed without eEmc.

We compared the pattern of EMG activity during step-like movements generated spontaneously in the cage (Figure 1E) to when the rat was stepping bipedally on a treadmill using the body weight support system (Figure 1F). Note that although the stimuli imposed did not induce synchronized (time locked to stimulation pulses) motor responses with each individual stimulus (Figure 1C), it was sufficient to enable a higher level of EMG activity in the TA and soleus and to produce motor responses that were asynchronous (not time locked to stimulation pulses) as occurs in the intact state (Figure 1D and E). Also note that there is a greater level of synchronous activity during treadmill stepping (Figure 1F) than during spontaneous cage activity (Figure 1E).

**Figure 2:**
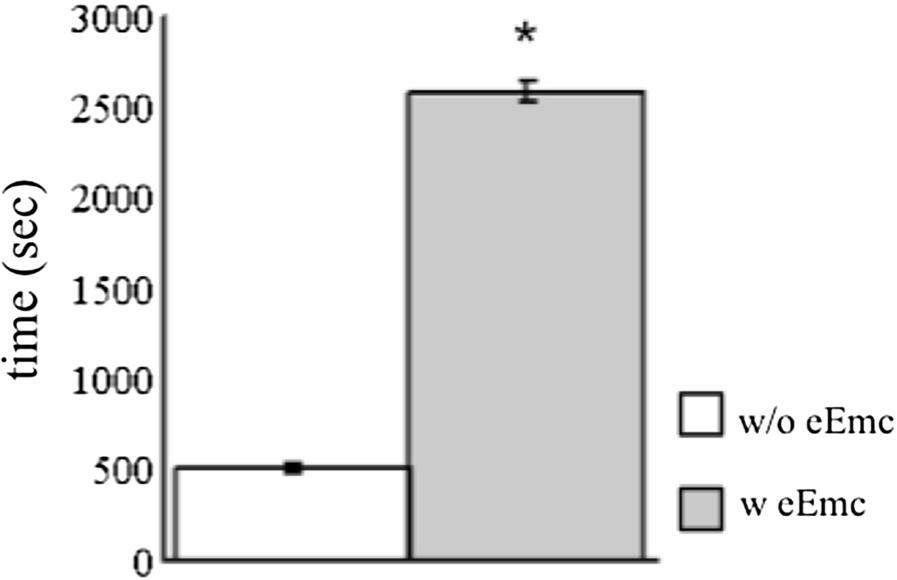
**Total activity time with and without eEmc.** Mean (±SEM, n = 4) duration of spontaneous cage activity during the 6-hr recording period with and without eEmc. *, significantly different from without eEmc at P < 0.05.

**Figure 3:**
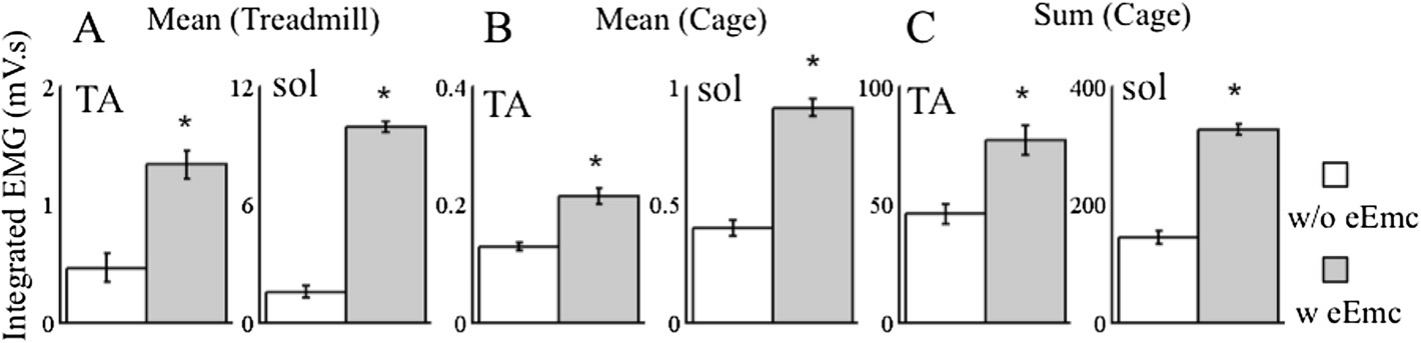
**Integrated EMG during spontaneous cage activity and treadmill locomotion. (A)** Integrated EMG during body weight supported treadmill stepping at 13.5 cm/sec for 1 min, **(B)** integrated EMG per min for the TA and soleus (sol) during the 6-hr recording period in the cage, and **(C)** sum of the integrated EMG during the 6-hr recording period in the cage for the TA and soleus muscles without and with eEmc. Values are mean ± SEM for 4 rats. *, significantly different from without eEmc at P < 0.05.

The total amount of time that the rats were active during these recordings was ~5-fold higher in the presence compared to the absence of eEmc, i.e., ~2500 sec or ~12% of the time vs. ~500 sec or ~2.5% of the time (Figure 2). The mean integrated EMG (Figure 3B) and summed integrated EMG (Figure 3C) for both the TA and soleus muscles during the 6-hr recording periods of spontaneous cage activity were significantly higher in the presence than in the absence of eEmc. To provide some point of reference regarding these increases in EMG activity with stimulation, the large differences in the mean integrated EMG in both muscles studied with and without eEmc when the rats were stepping on a treadmill are shown (Figure 3A). Furthermore, the amount of activity during the six hours of spontaneous cage activity was equivalent to ~33 minutes of stepping on the treadmill with eEmc compared to ~15 minutes without stimulation.

**Figure 4:**
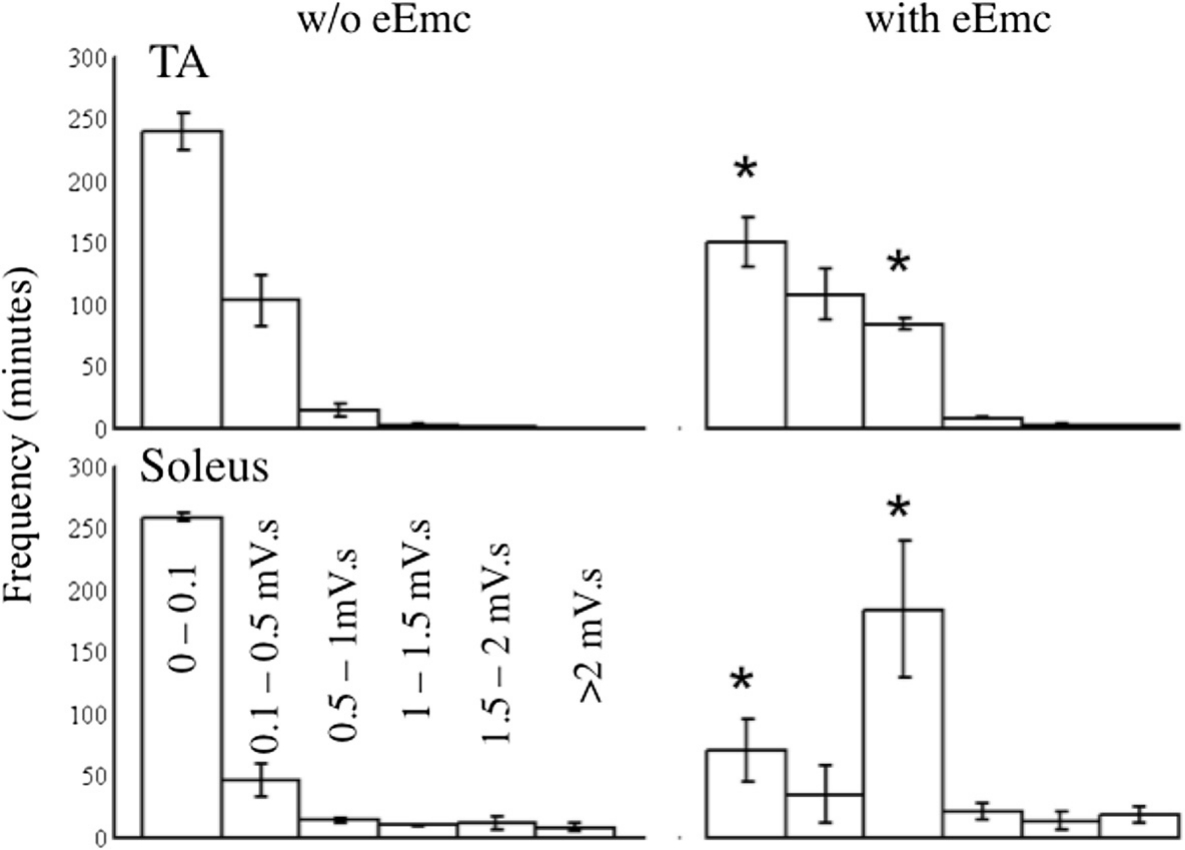
**Frequency distribution of integrated EMG.** Frequency distribution of the mean (±SEM, n = 4 rats) integrated EMG amplitudes for the TA and soleus with and without eEmc during the 6-hr recording period in the cage expressed in one-min bins. *, significantly different from the corresponding bin without eEmc at P < 0.05.

**Figure 5:**
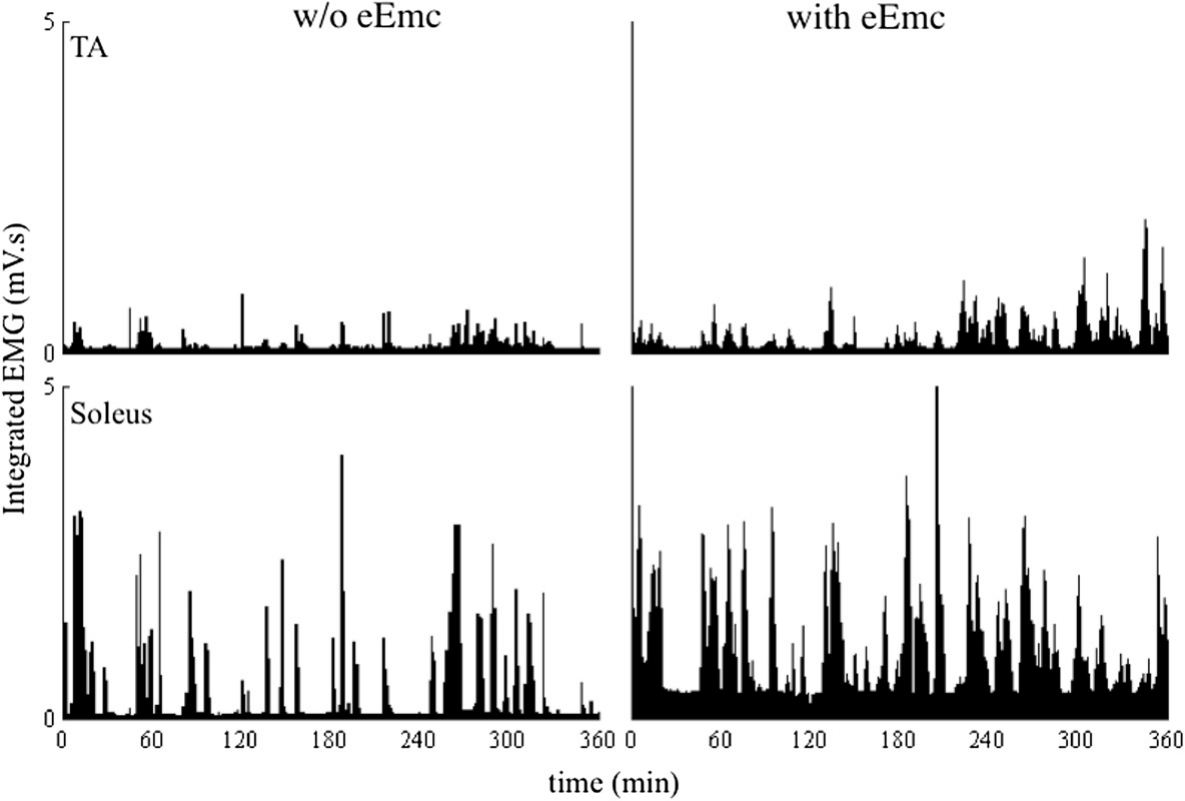
**Average integrated EMG with and without eEmc.** Mean (±SEM) frequency of occurrence of different ranges of integrated EMG amplitudes with and without eEmc during the 6-hr recording period of cage activity expressed in one-min bins.

**Figure 6:**
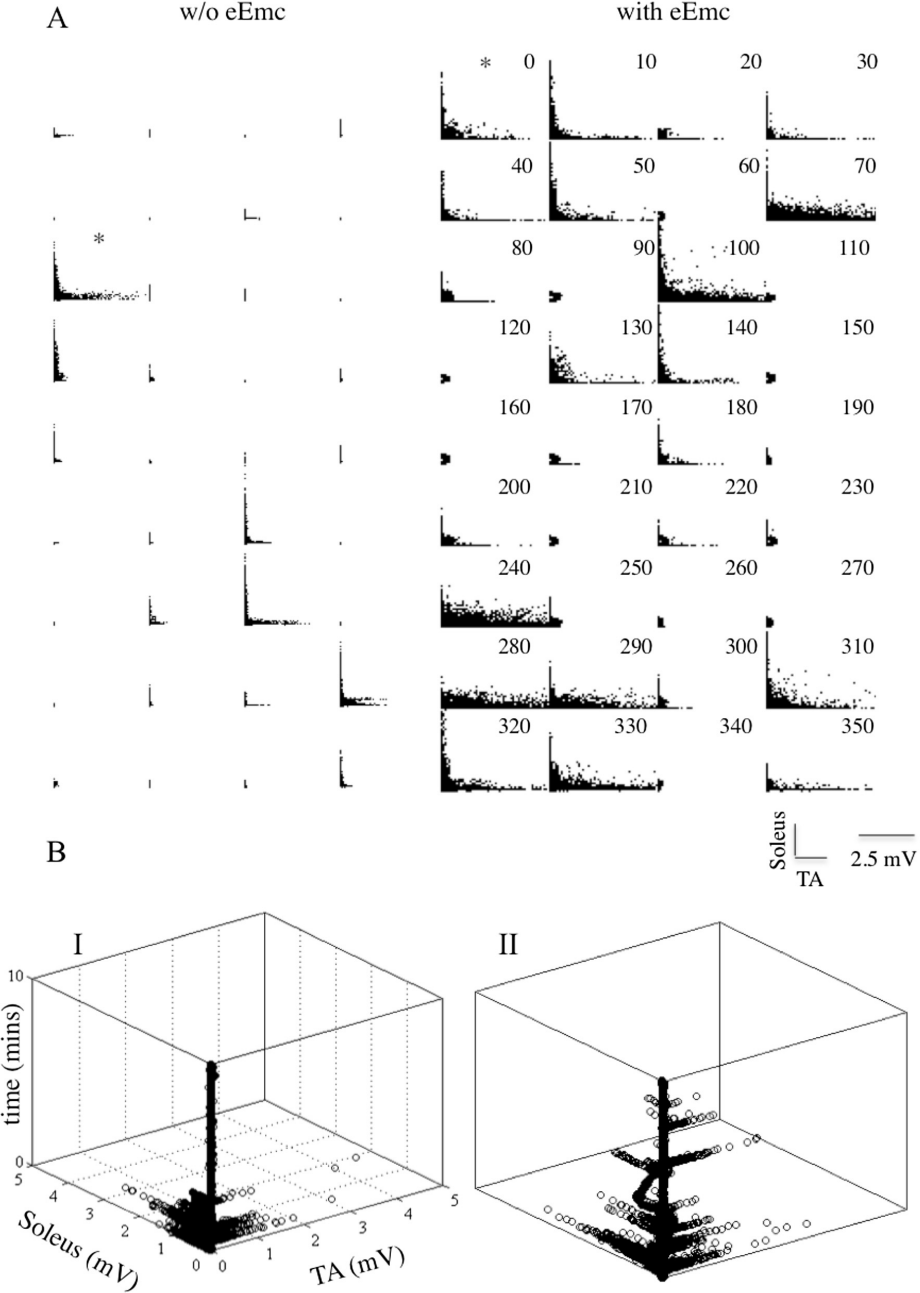
**JPD plots from a single animal throughout the 6 hours with and without eEmc. (A)** Joint probability distribution plots showing the relationship between the soleus and TA activity expressed in 10-min bins during the 6-hr recording period for a representative spinal rat. The 6-hr recording occurred during the dark period (8:00 pm to 2:00 am), i.e., the active period of the rats. **(B)** The incidence of occurrence of different joint probability distributions for 10 min of activity without (I) and with (II) eEmc. The asterisks in **(A)** identify the two bins being compared in **(B)**, without eEmc (I) and with eEmc (II). Note the lack of consistent alternating flexor-extensor activation without compared to with eEmc.

There was a larger number of one-min bins with relatively high levels of integrated EMG activity with than without eEmc distributed across the 6-hr recording period for both the TA and soleus (Figure 4). Differences in the frequency distributions of EMG amplitudes with and without eEmc also were evident (Figure 5). Higher EMG amplitudes were observed more frequently in both the TA and soleus in the presence of eEmc. There was greater evidence of reciprocal coordination between the TA and soleus muscles with than without eEmc across the 6-hr recording period (Figure 6A). The level of EMG amplitude modulation was greater in the TA than the soleus and with this increased occurrence of higher amplitudes in the TA there was clearly a higher incidence of co-contraction between the TA and soleus muscles without eEmc. In addition, instances showing apparent reciprocal activity without eEmc had fewer and less robust alternating patterns (Figure 6B I) compared to those observed with eEmc (Figure 6B II).

## Discussion

Spinal circuits controlling stepping and standing after a spinal cord injury can be improved by practicing those tasks, i.e., increasing the activation of those circuits [17,18]. In the present study we show that there is a minimal amount of spontaneous activity in the sensorimotor circuits that can facilitate standing and stepping after a mid-thoracic spinal cord transection in adult rats. eEmc below the level of the lesion, enhanced the amount of spontaneous activity several-fold (Figure 3 and Figure 5) and resulted in more robust stepping-like and partial weight-bearing standing activity (Figure 1D and E). If the spontaneous activity can be enhanced with eEmc, this in effect would suggest a 'self-training’ phenomenon. This effect would be consistent with the observation that independent, full weight-bearing standing can be initiated “voluntarily” and sustained in humans with complete paralysis in the presence of eEmc at an intensity that, in itself, induces little or no direct motor responses [7].

Does the elevated motor activity observed with sub-threshold spinal cord stimulation reflect some level of “voluntary” control? The report that a completely paralyzed human subject can regain the ability to stand under the influence of eEmc [7] raises the question as to whether this can be considered to be “voluntarily” initiated either indirectly or via some “reflex” mechanism. There are no “reflexes” described, however, that have the motor output features performed by the paralyzed subject noted above nor the rats in the present study. While there are no widely accepted criteria for describing if a task is performed 'voluntarily’ , the human subjects acquired the ability to initiate and sustain standing on command. Whether this could be viewed as being either a “voluntary” or an “automated” response, the subjects were able to volitionally position the upper body in a manner that increased weight bearing on the lower limbs with a critical level of eEmc. This, in turn, engaged the proprioceptive input to the spinal cord from the hindlimbs resulting in more weight-bearing activity, essentially as it seems to have been the case in the rats. To what extent can one routinely and voluntarily engage proprioception to perform a motor task? We propose that the observations in the spinal rats in the present study parallel the human data in that the rats increased their cage activity levels in the presence of sub-motor threshold stimulation intensities (Figures 2, 4 and 5). They were more active and mobile because the spinal networks were placed in a state of higher “readiness”, making it more feasible to “volitionally” engage the postural and locomotor circuits when the rat chose to be mobile. Given that proprioception can initiate and control a wide range of postural and locomotor tasks, it seems feasible that the elevated activity in the presence of eEmc occurred as a result of the intent of the rats to be mobile as reported previously [19].

The experiments in both rats [20] and humans [7,8] were designed to engage the “paralyzed circuits” during a specific training-rehabilitation time period in the presence of stimulation. Since the level of stimulation necessary to achieve the results noted above appeared to have little or no recognizable direct motor or behavioral effects on the animal or human subjects, we tested the hypothesis that sustained sub-threshold levels of activity in the normal cage environment would result in greater spontaneous activity among those spinal circuits that generate and control standing and stepping in rats. The implications of these observations are that the training effects induced via formal motor rehabilitation sessions could be greatly amplified during periods of routine daily activity enhanced by eEmc. These results now raise the question as to whether a general increase in activity as observed herein will result in improved standing and stepping ability compared to rats not stimulated, especially given the issue of the specificity of training. In this light, recent findings by Garcia-Alias et al. [21] demonstrate that rats that are housed in an enriched environment after a spinal cord injury are more active and perform significantly better in reaching and locomotor tasks than those housed in standard cages. In addition, we have studied the effects of one stimulation paradigm and it is highly likely that other stimulation paradigms may produce more robust task-specific effects.

The spinal rats in the present study were more spontaneously active with than without eEmc, even though they were housed in standard cages. It seems likely that a combination of eEmc and an enriched housing environment would result in even greater levels of spontaneous activity, particularly in rats that are completely paralyzed. Issues related to the type and intensity of the activity performed by a spinal cord injured patient (or animal) during the prolonged daily periods without any formal rehabilitation treatment (most likely >23 hrs) have come to the forefront only recently. Even in normal humans [22] and animals [23] 80-90% of the daily activity occurs at very low levels of activation of almost all motor pools. With the ability to carefully quantify muscle and body activity, surprising results have been reported in how daily activity levels change when uninjured individuals begin physical training [22].

The critical questions raised now are whether the effect of epidural stimulation alone or in combination with an enriched environment would result in improved performance of reaching, standing, and locomotion and how much and what type of spontaneous activity is sufficient to enhance each of these motor tasks. Can motor performance be improved after severe paralysis by enabling the spinal circuitry during routine daily activities in the home in addition to the specific training that occurs during structured rehabilitation sessions? The spontaneous activity that may occur in a wide range of sensorimotor pathways may result in progressive improvement in specific tasks requiring fine motor control of the hands or in postural and locomotor functions, particularly if the same motor pathways are engaged as they are during scheduled rehabilitative sessions.

## Conclusions

In the present study we demonstrate that that there is an enhanced amount of spontaneous activity in the sensorimotor circuits that can facilitate standing and stepping after a mid-thoracic spinal cord transection in adult rats using chronic subthreshold eEmc below the level of the lesion suggesting a novel 'self-training’ phenomenon.

## Abbreviations

eEmc: Electrical enabling motor control; TA: Tibilias Anterior; Sol: Soleus.

## Competing interests

The authors report no competing interest.

## Authors’ contribution

PG, JC and MR performed the experiments and analyzed the data. HZ and RRR performed the surgeries. PG, PS, GGA, YG, RRR and VRE interpreted the data. PG, YG, RRR and VRE wrote the manuscript. All authors read and approved the final manuscript.

## Supplementary Material

Additional file 1 **Video 1.** Representative animal without eEmc demonstrating the lack of hindlimb locomotion and unsuccessful attempt at bipedal standing. Click here for file

Additional file 2 **Video 2.** Representative animal with eEmc demonstrating the alternating hindlimb locomotion like activity during forelimb driven locomotion and bipedal standing with partial weight bearing using the sides of the cage as support. Click here for file
